# Respective influence of veterinarians and local institutional stakeholders on the event-driven surveillance system for bovine brucellosis in France

**DOI:** 10.1186/s12917-015-0499-1

**Published:** 2015-08-01

**Authors:** Anne Bronner, Eric Morignat, Didier Calavas

**Affiliations:** ANSES-Lyon, Unité Epidémiologie, 31 avenue Tony Garnier, 69364 Lyon, Cedex 07, France

**Keywords:** Veterinary, Institutional stakeholders, Bovine abortion, Brucellosis surveillance, Event-driven surveillance

## Abstract

**Background:**

The event-driven surveillance system for bovine brucellosis implemented in France aims to ensure the early detection of cases of bovine brucellosis, a disease of which the country has been declared free since 2005. It consists of mandatory notification of bovine abortions by farmers and veterinarians. However, as underlined by a previous qualitative study, several factors influence the decision-making process of actors in the field. This process is particularly influenced by the level of cooperation between institutional stakeholders in their *département* (a French *département* being an administrative and territorial unit), veterinarians and farmers. In this context, the objectives of this study were 1) to quantify the respective influence of veterinarians and all local institutional stakeholders on the proportion of notifying farmers and identify which actors have most influence on farmers’ decisions; 2) to analyse whether the influence of veterinarians is correlated with that of local institutional stakeholders.

**Results:**

In addition to factors relating to the farm itself (production type and herd size), the proportion of notifying farmers was influenced by the number of veterinarians per practice and the veterinary practice’s membership of a technical association. This proportion was also influenced by unknown factors relating to the veterinary practice and, to a lesser extent, the *département* in which the farm was located. There was no correlation between variability in the proportion of notifying farmers among veterinary practices per *département* and the effect of the *département* itself.

**Conclusion:**

To our knowledge, this is the first study to quantify the influence of veterinarians and local institutional stakeholders on the notification process for a mandatory disease. In addition to carrying out regulatory interventions, veterinarians play a major role in encouraging farmers to participate in the surveillance systems. The results of this study, combined with a previous qualitative study, shed light on the need to consolidate the involvement of veterinarians and local stakeholders in the organisation of surveillance by national institutional bodies.

## Background

Event-driven surveillance, or passive surveillance, refers to observer-initiated provision of animal health related data (e.g. voluntary notification of suspect disease). In France, the goal of the event-driven surveillance system for bovine brucellosis is to ensure the early detection of bovine brucellosis, a disease of which the country has been declared officially free since 2005. The system relies on the mandatory notification of each bovine abortion, defined by the French Rural Code as the expulsion of a foetus or a calf, stillborn or dying within 48 h of birth [1]. According to national regulations, farmers must contact their authorised veterinarian for each bovine abortion. This veterinarian, chosen by the farmer, is mandated by the veterinary services to carry out regulatory interventions such as notification of suspected clinical cases or collection of samples. The veterinarian must then report the abortion, collect data (such as the female identification number (ID), stage of pregnancy and date of abortion) and take a blood sample from the aborting female to test for *Brucella* spp. The event-driven surveillance system for bovine brucellosis is managed by local veterinary services, which are in charge of implementing surveillance on a *département* scale (a French *département* being an administrative and territorial unit with a mean surface area of 5,800 km^2^), following up on laboratory results, and paying for veterinarians’ visits and laboratory tests for brucellosis.

Apart from these regulatory measures, the GDS (*Groupement de Défense Sanitaire*, a *département*-level association of stock farmers addressing animal health issues) in some *départements* have developed their own diagnostic protocol for enzootic abortive diseases (such as Q fever, neosporosis and bovine viral diarrhoea). These protocols aim to provide technical support to veterinarians for identifying the causes of abortions, considering the direct financial losses for farmers. Diagnostic tests based on these protocols are partly funded by the GDS in some *départements*. Training courses and information for veterinarians on abortion diagnoses are supported by GTVs (*Groupement technique vétérinaire*, a technical veterinary association found in each *département*). Although there is a GDS and a GTV in each *département*, due to historical considerations, the proportion of GDS members is not far from 100 % whereas only about 43 % of veterinary practices who carry out regulatory interventions in cattle herds are GTV members.

However, despite national regulations and the importance for farmers themselves in preventing public and animal health risks related to brucellosis and other abortive diseases, the under-reporting of abortions is a major limitation to the brucellosis event-driven surveillance system [2]. By using a qualitative study based on semi-structured interviews of farmers and their veterinarians, we highlighted factors influencing the decision-making process of farmers and veterinarians [3]. This qualitative study relied on “purposive sampling” [4], and participants were chosen in order to cover a variety of herd characteristics (for farmers) and attitudes towards their duty to report abortions. Therefore, the influence of these specific factors could not be quantified in this study. But this analysis showed that four main themes influence the decision-making process of farmers and veterinarians: 1) the perceived risk of brucellosis and other abortive diseases; 2) the definition of a suspected case of brucellosis and other abortive diseases adopted by actors in the field, which is less sensitive than the mandatory definition; 3) the cost-benefit analysis conducted by actors, taking into account regulatory and health aspects, economic and financial losses, technical and practical factors; 4) the level of cooperation between veterinary services, GDSs, GTVs, authorised veterinarians and farmers in each *département* [3].

Based on this qualitative study, we assumed that the proportion of notifying farmers (i.e. the ratio of the number of farmers who reported at least one abortion to the total number of farmers) was influenced by veterinarians and the different local institutional stakeholders. Some studies have quantified variability in the attitudes and perceptions of veterinarians towards specific diseases [5], biosecurity measures [6] or the control of enzootic diseases [7]. In human health, several epidemiological studies have demonstrated considerable variations in the notification practices of general practitioners [8, 9], and these might exist in the animal health sector as well. To our knowledge, however, no studies have so far quantified the influence of veterinarians on the implementation of a mandatory surveillance system. Due to a lack of data, the overall involvement of local institutional stakeholders in the event-driven surveillance system for bovine brucellosis was studied by using the *département* as a proxy.

In this context, the objectives of the present study were 1) to quantify the respective influence of veterinarians and all local institutional stakeholders (veterinary services, GDSs, GTVs) on the proportion of notifying farmers and identify which actors have most influence on farmers’ decisions; 2) to analyse whether the influence of veterinarians is correlated with that of local institutional stakeholders.

## Material and method

### Data sources and study population

For every cattle farm, information about the location (*département*), animals (identification number, birth date, sex and breed), and animal movements (herd identification number, date, reason for entry and exit) were extracted from the French National Cattle Register. Abortion notifications and data on veterinarians were extracted from the French national animal health information database (SIGAL, *Système d’information de la Direction générale de l’alimentation*). Due to administrative reasons (the veterinary services reimburse veterinarians’ visits in the event of abortion to veterinary practices and not to veterinarians themselves), data on veterinarians refer to veterinary practices and not to individual veterinarians.

The study focused on abortions reported from 1 July 2011 to 30 June 2012. It included all *départements* that had reported at least one abortion per year since 2010, and cattle herds where at least one calving was recorded and where the same veterinary practice carried out regulatory interventions over the study period. A cattle herd was characterised by its *département*, size, production type, the veterinary practice mandated by veterinary services to carry out regulatory interventions in this herd over the study period, the number of abortions reported and the dates of veterinarian visits. Herd size was calculated as the mean number of females over 24 months of age per week. Five production types were defined according to the breed and number of calvings over the study period (Table 1). A veterinary practice was characterised by its national registration number, the size of its clientele and its GTV membership. The clientele size was calculated as the mean number of females over 24 months of age per week recorded in farms where a veterinary practice carried out regulatory interventions. A *département* was characterized by its proportion of GTV members (i.e. the ratio of the number of veterinary practices being a GTV member to the total number of veterinary practices carrying out regulatory interventions in cattle herds).

**Table 1:** Typology of the production type of cattle herds

Production type	Definition
Beef	Herds with more than 10 calvings from beef females and fewer than 10 calvings from dairy females.
Dairy	Herds with more than 10 calvings from dairy females and fewer than 10 calvings from beef females.
Mixed	Herds with more than 10 calvings from dairy females and more than 10 calvings from beef females.
Small herd	Herds with fewer than 10 calvings from dairy females and fewer than 10 calvings from beef females, with a mean number of females over 24 months of age held per week of less than 10, and from which fewer than 10 males were sent to slaughterhouse.
Other	Herds with fewer than 10 calvings from dairy females and fewer than 10 calvings from beef females, with a mean number of females over 24 months of age held per week above 10, and/or from which more than 10 males were sent to the slaughterhouse.

In accordance with national regulations, personal data on farmers and veterinarians are collected through the bovine brucellosis surveillance system. The data used in this study are not freely available because of legal restrictions. Their access was authorised by the French Ministry of Agriculture in the context of this study, provided the results remain anonymous.

### Modelling the proportion of notifying farmers

The proportion of notifying farmers was analysed with a logistic regression model that included covariates as follows:

$$ \begin{array}{l}\mathrm{Logit}\left({\mathrm{p}}_{\mathrm{i}\mathrm{jk}}\right)={\mathrm{D}\acute{\mathrm{e}} \mathrm{partement}}_{\mathrm{j}}+{\mathrm{Veterinarian}}_{\mathrm{k}}+\mathrm{Production}\ {\mathrm{type}}_{\mathrm{i}}+{\mathrm{Size}}_{\mathrm{i}}+{\mathrm{Size}}_{\mathrm{k}}\\ {}+\mathrm{Number}\_{\mathrm{vet}}_{\mathrm{k}}+{\mathrm{GTV}}_{\mathrm{k}}+\mathrm{Prop}\ \_{\mathrm{GTV}}_{\mathrm{j}}\end{array} $$

p_ijk_ was the proportion of notifying farmers holding a cattle herd located in a *département j* with production type and size *i*, for which the veterinarian practice *k* carries out regulatory interventions. The location of the herd (*département*) and the veterinary practice were included as crossed random effects [10]. Indeed, some veterinary practices carry out interventions in several *départements* and thus, their influence on the proportion of notifying farmers can interact across different *départements*.

The other covariates were: 1) with respect to farms, the production type “Productiontype_i_” (dairy, beef, mixed herds, small herds, other production type, see Table 1) and herd size “Size_i_” (with categories based on the distribution of this covariate); 2) with respect to veterinary practices, the clientele size “Size_k_” (with categories based on the distribution of this covariate), the number of veterinarians working in the veterinary practice “Number_vet_k_” (with two categories: one, or more veterinarians), GTV membership of the practice “GTV_k_” (a binary covariate); 3) with respect to the *département*, the proportion of GTV members “Prop_GTV_j_” (with categories based on the distribution of this covariate).

We adopted a backward model selection process, which involves starting with all candidate covariates, testing the deletion of each covariate using the Akaike Information Criterion (AIC) [11], deleting them if the model was improved by doing so, and repeating this process until no further improvement was possible. Two-factor interactions were tested, among farm-level covariates and veterinarian-level covariates, respectively. We checked model fit by studying quantile-quantile plots of quantiles of residuals as proposed by [12], assuming the fitted model is the true model versus the actual quantiles of residuals from the fitted model. In order to quantify the respective influence of veterinarians and institutional stakeholders on the proportion of notifying farmers, variability in the proportion of notifying farmers among *départements* and among veterinary practices was studied based on the distribution of the random effects estimated for each *département* (“*Département*_*k*_”) and veterinary practice (“*Veterinarian*_*k*_*”)* respectively. The odds ratio (OR) of the effect of a *département j* or a veterinary practice *k* was calculated as exp(*Département*_*j*_) or exp(*Veterinarian*_*k*_) respectively.

In order to analyse whether the influence of veterinarians was correlated with the influence of institutional stakeholders, we calculated the correlation between the variability in the proportion of notifying farmers among veterinary practices in *département j* and the effect of this *département j* on the proportion of notifying farmers (“*Département*_*j*_*”)* using the Pearson correlation test. The underlying assumption was that a highly coordinated brucellosis event-driven surveillance system by local institutional stakeholders would be related to harmonised practices among farmers and veterinarians. According to this hypothesis, the variability in the proportion of notifying farmers among veterinary practices would tend to decrease as the *département* proportion of notifying farmers increases. For each *département j*, the variability in the proportion of notifying farmers among veterinary practices was computed as the component of two terms: 1) the inter-individual variation among veterinary practices, estimated as the variance of the distribution of the mean effect *Veterinarian*_*k*_ estimated for veterinarian practices located in this *département*; 2) the intra-individual variation, estimated as the mean standard errors of *Veterinarian*_*k*_ estimated for veterinarian practices located in this *département* (using the function *se.ranef()* in R). The statistical analyses were performed with R [13] and the lme4 [14] and arm [15] packages.

## Results

### Population characteristics

The study population included 181,531 cattle herds in 82 *départements* and the 1,894 related veterinary practices: 33.7 % (n = 61,255) were beef cattle, 28.8 % (n = 52,205) vdairy cattle, 8.7 % (n = 15,629) mixed herds, 20.2 % (n = 36,748) were small cattle herds and 8.6 % (n = 15,694) of another production type. Herd size varied from one to 708 females-week (median value: 42). Veterinarians worked alone in 31 % (n = 590) and with colleagues in 69 % (n = 1,304) of veterinary practices respectively. More than half (55 %, n = 1,054) of veterinary practices carried out regulatory interventions in one *département*, 33 % (n = 626) in two *départements*, 11 % (n = 193) in three *départements* and the others (n = 21) in four or five *départements*. Clientele size varied from one to 39,897 females-week (median value: 3,047). Overall, 716 (38 %) of veterinary practices were a GTV member and the proportion of GTV members varied between 6 and 76 %, depending on the *département* (median value: 43 %). For 23.6 % (n = 447) of veterinary practices, no farmers reported any abortions over the study period.

### Factors influencing the proportion of notifying farmers

The quantile-quantile plot suggested there were no major departures from the model assumptions. The final model included all the covariates tested except two (clientele size and proportion of GTV members in the *département*). Interactions did not significantly improve the model. Odds ratios and variances for the *départements* and veterinary practices are displayed in Table 2. For *départements*, 95 % of the random effects varied from −0.64 to 0.62 (i.e. OR varying between 0.52 and 1.87). For veterinary practices, 95 % of the random effects varied from −0.77 to 0.95 (i.e. OR varying between 0.46 and 2.59). The correlation between the variability in the proportion of notifying farmers among veterinary practices in *département j* and the effect of this *département j* on the proportion of notifying farmers was not significant (p = 0.9).

**Table 2:** Odds ratio estimates from the logistic regression model

Variable		Odds ratio [95 % CI]
Herd production type	Beef	1
	Dairy	3.15 [3.03 - 3.27]
	Mixed	2.0 [1.9 - 2.09]
	Small herd	0.13 [0.12 - 0.15]
	Other	0.40 [0.37 - 0.44]
Herd size	<50	1
	[50-100[	1.95 [1.88 - 2.01]
	≥100	3.10 [2.97 - 3.23]
Veterinary practice	One veterinarian	1
	More than one veterinarian	1.12 [1.03 - 1.23]
Membership of a technical veterinary association^a^	No	1
	Yes	1.37 [1.27 – 1.47]
“*Département”* variance		0.13
“Veterinary practice” variance		0.32

95 % confidence intervals are mentioned in square brackets and values in bold type indicate significant differences (i.e. confidence interval not including 1.00)

^a^namely Groupement technique vétérinaire (GTV)

## Discussion

By analysing the proportion of farmers who participated in the French event-driven surveillance system for bovine brucellosis in 2011/2012, we intended to quantify the respective influence of veterinarians and all local institutional stakeholders (local veterinary services, GDSs and GTVs) on the implementation of this surveillance system. The proportion of notifying farmers was strongly influenced by farm factors (production type and herd size), with an OR up to 3.15. Factors at the veterinary practice level had a significant but lower effect, with an OR estimated at 1.12 and 1.37 for respectively the number of veterinarians and GTV membership, and 95 % of the OR estimated for other unknown factors ranged from 0.46 to 2.59. Lastly, factors at the *département* level had the lowest effect: their influence was taken into account by a single random effect, with 95 % of the estimated OR varying between 0.52 and 1.87. The variability in the proportion of notifying farmers among veterinary practices at a *département* scale was not correlated with the effect of the *département*.

### Limitations of the study

Our analysis focused on abortion notification although the notification process involves abortion occurrence, abortion detection and abortion notification. Indeed, we assumed that veterinarians and local institutional stakeholders influence abortion notification in particular.

In order to quantify the overall influence of local institutional stakeholders on the proportion of notifying farmers, we used the *département* as a proxy. We are aware that such an approach prevents us from differentiating the specific influence of local institutional stakeholders from other factors that actors in the field actors could share at the *département* level. In particular, the history of brucellosis in a *département* might influence their perception of brucellosis risk, and thus their willingness to report abortions. However, such an influence was not identified during our qualitative study [3]. Part of the variability among *départements* may also be due to farming practices, which differ between areas of production and might influence the ability of farmers to detect abortions. But as the production type was included in the final model, we assumed these effects were low in comparison to the influence of local institutional stakeholders. Therefore, we assumed that the *département* effect could be interpreted as an effect mainly due to the different local institutional stakeholders.

Due to a lack of available data, variations in the proportion of notifying farmers were studied in relation to the veterinary practice and not the individual veterinarian. We are aware that studying the influence of each veterinarian would have revealed a greater variability in the proportion of notifying farmers among veterinarians. However, variations in the proportion of notifying farmers among veterinarians are likely to be lower when they work together than when they belong to different veterinary practices, as they are more likely to share common practices in the former case (including participation in the brucellosis event-driven surveillance system and the quality of data they collect). In any case, this hypothesis should be properly addressed.

### Influence of specific factors related to the veterinarian and *département*

Based on our study, the proportion of notifying farmers was higher among veterinary practices with more than one veterinarian than among veterinarians working alone. Veterinarians who work with other colleagues might be more inclined to report abortions than those who work alone: they can divide up tasks and thus have less need for giving priority to emergencies over regulatory interventions than veterinarians who work alone. They can also motivate each other to report abortions and discuss matters with colleagues should they have technical difficulties in identifying abortion aetiology.

Based on our study, veterinarians who are GTV members are more prone to report and/or to encourage farmers to report abortions than others. Those veterinarians are likely to be committed to developing technical expertise, and they find in this membership the way to maintain and develop this expertise (e.g. training courses and/or information). As revealed by the previous qualitative study, some veterinarians report abortions because they have a technical interest in doing so [3]. On the contrary, veterinarians who are not GTV members might lack the technical skills needed to identify the cause of abortion, which is considered difficult due to the wide range of potential pathogens, the ubiquity of pathogens such as Q fever or salmonellosis [16], and the lack of knowledge about differential diagnosis protocol [3].

Our study did not reveal any influence of clientele size and of the proportion of GTV members on a *département* scale on the proportion of notifying farmers. Cliente size was included as a proxy of the proportion of veterinarians’ activity dedicated to cattle herds (unavailable data), assuming that the higher this proportion was, the more prone veterinarians were to participate in bovine disease surveillance. The proportion of GTV members on a *département* scale was assumed to reflect the involvement of local GTVs in coordination and technical support of veterinarians. The absence of effect of these two covariates might be due to the fact that they did not correctly reflect the veterinarians’ activity and the activity of local GTVs, respectively. Moreover, the fact that most information and training courses are organised and carried out at nationally could explain the weak influence of local GTVs on veterinarians’ practices.

### Respective influence of farmers, veterinarians and local institutional stakeholders

Based on the quantitative results of our study, the proportion of notifying farmers was influenced, in this order, by production type and herd size, veterinarians and, to a lesser extent, by local institutional stakeholders. These results reflect the respective role of each actor on the abortion notification process.

Farmers are key actors who detect and call for a veterinarian in the event of an abortion, and they are strongly influenced by mechanical and practical issues. The number of abortions, and thus the probability for a farmer to report abortions, increases with herd size. As underlined by the previous qualitative study, beef cattle farmers face difficulties in detecting aborting females and catching them when they are at pasture for a serological analysis [3].

Veterinarians are likely to have a greater influence on the decisions of farmers to call them for an abortion than institutional stakeholders, as they have frequent interactions with farmers. When veterinarians are keen to report abortions, they can have a strong effect on farmers. But their own decision to report abortions and to promote the importance of abortion notification among farmers depends on several individual factors, as highlighted by the previous qualitative study [3].

Beyond the role of veterinarians, our study underlined the role local institutional stakeholders could have on the involvement of veterinarians and farmers in the mandatory abortion surveillance system, even if this role was smaller than that of veterinarians. Indeed, the implementation of the event-driven surveillance system for brucellosis and an abortion diagnosis protocol might differ among institutional stakeholders. Coordination of veterinarians, including technical support and information on the results of the mandatory surveillance systems, depends greatly on local veterinary services. Likewise, the abortion diagnosis protocols provided by some GDSs might encourage actors in the field to test aborting cows for diseases other than brucellosis and thus to report abortions. In some *départements*, institutional stakeholders provide veterinarians with sampling material (kits with a vaginal swab, dry or EDTA tubes, etc.) and manage the shipment of samples to the laboratory. But despite some efforts, local institutional stakeholders face difficulties in encouraging farmers and veterinarians to report abortions, which might explain their weak influence on the proportion of notifying farmers identified in our study.

Besides, based on our results, the influence of veterinarians is not correlated with the influence of local institutional stakeholders. Veterinarians report or do not report abortions regardless of the level of surveillance system coordination by local institutional stakeholders: some are prone to report abortions in *départements* with a low proportion of notifying farmers whereas others are not, even if they are located in *départements* with a high proportion of notifying farmers.

### Prospects for improving the coordination of veterinarians and institutional stakeholders

Based on the results of this study, there is a need for local institutional stakeholders to standardise the activity of veterinary practices in order to have a more effectively coordinated and consistent disease surveillance system. Veterinarians act as the interface between veterinary services, GDSs, GTVs and farmers. Besides carrying out regulatory interventions, they play a major role in encouraging farmers to participate in these surveillance systems. This role should be promoted locally by taking into account their difficulties and expectations. Nationally, a diagnosis protocol for abortive diseases was recently drawn up based on scientific requirements for sampling and laboratory analyses. Improving the coordination of veterinarians by institutional stakeholders providing technical support, training and information on the results of the abortion diagnosis protocol (in addition to the results from the mandatory surveillance system) is also expected to increase their participation in the surveillance system [17]. These coordination actions should be standardised across *départements* by national institutional bodies.

## Conclusions

To our knowledge, this is the first study to quantify the respective influence of veterinarians and local institutional stakeholders on the notification process of a mandatory disease. In addition to carrying out regulatory interventions, veterinarians play a major role in encouraging farmers to participate in the surveillance systems. However, improving their involvement is a challenge, considering their number, heterogeneous practices, knowledge and attitudes. The results of this study, combined with a previous qualitative study [3], shed light on the need to consolidate the involvement of veterinarians and local institutional stakeholders in the organisation of surveillance by national institutional bodies. Such initiatives would require special staff, but would also increase actors’ participation in the surveillance system.

## References

[1] Anonymous (2003). Article R. 223–79 du Code rural et de la pêche maritime.

[2] Bronner A, Hénaux V, Vergne T, Vinard J-L, Morignat E, Hendrikx P (2013). Assessing the mandatory bovine abortion notification system in France using unilist capture-recapture approach. Plos One.

[3] Bronner A, Hénaux V, Fortané N, Hendrikx P, Calavas D (2014). Why farmers and veterinarians do not report all bovine abortions, as requested by the clinical brucellosis surveillance system in France?. BMC Vet Res.

[4] Glaser B, Strauss A (1967). The discovery of grounded theory. Strategies for qualitative research.

[5] Genchi C, Bowman D, Drake J (2014). Canine heartworm disease (Dirofilaria immitis) in Western Europe: survey of veterinary awareness and perceptions. Parasit Vectors.

[6] Gunn G, Heffernan C, Hall M, McLeod A, Hovi M (2008). Measuring and comparing constraints to improved biosecurity amongst GB farmers, veterinarians and the auxiliary industries. Prev Vet Med.

[7] Higgings H, Huxley J, Wapenaar W, Green M (2014). Quantifying veterinarians' beliefs on disease control and exploring the effect of new evidence: a bayesian approach. J Dairy Sci.

[8] Day F, Sutton G (2007). General practitioner notifications of gastroenteritis and food poisoning: cause for concern. J Public Health.

[9] Figueiras A, Lado E, Fernandez S, Hervada X (2004). Influence of physicians' attitudes on under-notifying infectious diseases: a longitudinal study. Public Health.

[10] Baayen R, Davidson D, Bates D (2008). Mixed-effects modeling with crossed random effects for subjects and items. J Mem Lang.

[11] Burnham K, Anderson D (1998). Model selection and inference: a practical information - theroretic approach.

[12] Zuur A, Ieno E, Walker N, Saveliev A, Smith G (2009). Mixed effects models and extensions in ecology with R.

[13] R: A language and environment for statistical computing. R Foundation for Statistical Computing, Vienna, Austria, ISBN 3-900051-07-0 [http://www.R-project.org].

[14] Package lme4 [http://cran.r-project.org/web/packages/lme4/lme4.pdf].

[15] Package arm [http://cran.r-project.org/web/packages/arm/arm.pdf].

[16] Carpenter TE, Chriel M, Andersen MM, Wulfson L, Jensen AM, Houe H (2006). An epidemiologic study of late-term abortions in dairy cattle in Denmark, July 2000-August 2003. Prev Vet Med.

[17] Sawford K (2011). Animal health surveillance for early detection of emerging infectious disease risks.

